# Lung Cancer Attracts Greater Stigma than Other Cancer Types in Aotearoa New Zealand

**DOI:** 10.1155/2022/2183055

**Published:** 2022-08-26

**Authors:** Jess Godward, Benjamin C. Riordan, Taylor Winter, John C. Ashton, John Hunter, Damian Scarf

**Affiliations:** ^1^Department of Psychology, University of Otago, Dunedin, New Zealand; ^2^Centre for Alcohol Policy Research, La Trobe University, Melbourne, Australia; ^3^Department of Psychology, Victoria University of Wellington, Wellington, New Zealand; ^4^Department of Pharmacology and Toxicology, University of Otago, Dunedin, New Zealand

## Abstract

**Background:**

Lung cancer is the leading cause of cancer death in Aotearoa New Zealand, killing over 1,700 people each year. Despite the burden of lung cancer in Aotearoa New Zealand, the popular press has referred to it as the cancer type that no one talks about. Here, we investigate one factor that may contribute to this state of affairs: lung cancer stigma.

**Methods:**

Participants were university students and members of the general public. University students were recruited via an online experiment participation system in 2021. Members of the public were recruited via social media. All participants completed the Cancer Stigma Scale (CSS) for one of five cancer types (lung, cervical, breast, skin, or bowel). The CSS is a 25-item scale with six subscales: awkwardness, avoidance, severity, policy opposition, personal responsibility, and financial discrimination.

**Results:**

The mean age of participants was 24.3 (Standard Deviation = 10.4). Data from each subscale were submitted to an analysis of covariance (ANCOVA), with cancer type as a between-participant factor (5: lung, cervical, breast, skin, or bowel) and stigma as the dependent variable. Relative to most other cancer types, people were more likely to avoid someone with lung cancer, view interacting with someone with lung cancer as more awkward, and view people with lung cancer as being responsible for their condition.

**Conclusion:**

The Health Research Council of New Zealand recently funded the very first trial of lung cancer screening in Aotearoa New Zealand. The current study suggests that addressing stigma will be essential for the success of such programs, with stigma likely influencing those who engage in such trials.

## 1. Introduction

Lung cancer is the leading cause of cancer death in Aotearoa New Zealand, killing over 1,700 people each year [1]. Moreover, lung cancer continues to be the leading cause of cancer death among Māori (the Indigenous peoples of Aotearoa New Zealand) and displays the most marked and sustained ethnic disparities between Māori and non-Māori [2]. For example, the standardised rate difference (SRD) between Māori and non-Māori for lung cancer death is 23/100,000 [2]. To put this number in context, the SRD for colorectal cancer, the second leading cause of cancer death in Aotearoa New Zealand (∼1,200 deaths annually), is negligible at −1/100,000 [2].

Despite the burden of lung cancer in Aotearoa New Zealand, a recent news article called lung cancer deaths “the cancer disgrace that no one talks about” [3]. A recent analysis of Health Research Council of New Zealand funding also demonstrates that, on the basis of the number of lives it takes each year, lung cancer receives a markedly smaller share of cancer research funding than one might predict [4]. Similarly, a search of the Charities Register reveals that the sole lung-cancer-specific charity (Lung Foundation New Zealand) received just $56,412 in 2019, an amount that is dwarfed by donations for other cancer-specific charities (e. g., Leukaemia & Blood Cancer New Zealand: $7,565,558; Breast Cancer Foundation New Zealand: $6,953,552; Prostate Cancer Foundation of New Zealand: $1,591,265). These disparities between the burden of lung cancer and nonprofit organisation funding are not unique to Aotearoa New Zealand [5].

When asked about the potential reasons underlying the lack of public attention, funding, and donations, National Lung Cancer Working Group member Professor Ross Lawrenson hypothesized that the low survival rate meant that few people made the transition from patient to advocate [3]. Here, we investigate another factor that may help explain the current status of lung cancer treatment in Aotearoa New Zealand: lung cancer stigma. In the context of health, stigma is defined as “…a social process or related personal experience characterised by exclusion, rejection, blame, or devaluation that results from experience or reasonable anticipation of an adverse social judgment about a person or group identified with a particular health problem” [6]. The general framework for health-related stigma came from work on human immunodeficiency virus infection and acquired immunodeficiency syndrome (i. e., HIV/AIDS). For example, a common misconception was that someone with HIV/AIDS must have engaged in promiscuous sex and/or intravenous drug use [7]. As a result, people with HIV/AIDS were the focus of a great deal of stigma, with the public viewing them as being responsible for their condition. If we extend this example to lung cancer, stigma may develop due to the general public's belief that lung cancer is only caused by smoking [8]. In this view, lung cancer is viewed as being both preventable and self-inflicted [9].

To our knowledge, not only are there no quantitative studies on lung cancer stigma in Aotearoa New Zealand, but there is also little mention of its potential role in how we treat those with lung cancer. This is an issue for three primary reasons. First, if lung cancer stigma is common among the general public, then there is likely to be less political pressure or backlash when lung cancer drugs do not receive funding. Indeed, although the focus of some media coverage, Pharmac's decision to backtrack on funding Keytruda was met with relatively little outrage [10]. Second, data from the United States suggests that general practitioners (GPs) are not immune to lung cancer stigma, with a vignette study revealing that GPs are less likely to refer patients with lung cancer symptoms to specialist treatment [11]. Although an indirect measure, evidence of issues with primary care in Aotearoa New Zealand can be drawn from the fact that a large proportion of people with lung cancer are diagnosed after presenting to an emergency department [4, 12]. Finally, the stigma the general public and health professionals hold toward lung cancer directly impacts patients' mental health. If dealing with lung cancer was not difficult enough, lung cancer patients tend to internalise public stigma, leading to feelings of shame, guilt, anger, and self-blame [13].

### 1.1. Current Study

As a first step toward characterising lung cancer stigma in Aotearoa New Zealand, we investigate whether people with lung cancer are the recipients of higher levels of stigma than patients with other cancer types. Specifically, following Marlow et al.'s [8] work in the United Kingdom, we recruited a nonpatient sample of participants and had them complete the Cancer Stigma Scale (CSS). Participants completed the scale for one of five cancer types (lung, cervical, breast, skin, or bowel). We had a single hypothesis that people with lung cancer would be the targets of higher levels of stigma than people with cervical, breast, skin, or bowel cancer.

## 2. Method

### 2.1. Participants and Procedure

The majority of participants (78%) were students at the University of Otago, Aotearoa New Zealand, and were recruited online through an experiment participation system in 2021. The remaining participants were recruited via social media (e. g., Facebook). Student participants received course credit for participating. Participants recruited via social media received no reimbursement. In total, three hundred and forty-nine people participated in the current study (Mean Age = 24.2, Standard Deviation = 10.4; 275 females, 69 males, and 5 people identified as neither female nor male; Table 1). The majority of participants identified as New Zealand European (*n* = 255, 73.1%), followed by Māori (*n* = 32, 9.2%), Asian (*n* = 22, 6.3%), Pacific (*n* = 4, 1.1%), and other (*n* = 36, 10.3%). All participants provided informed consent before being randomly assigned to answer the CSS with regard to one of five cancer types (lung *n* = 72, cervical *n* = 66, breast *n* = 70, skin *n* = 70, or bowel *n* = 71; Table 1). The current study was approved by the University of Otago Human Ethics Committee (Reference: D21/193). An earlier version of the current manuscript is available as a preprint on PsyArXiv [14].

### 2.2. Measures

#### 2.2.1. Demographics

The demographics were charaterised on the basis of gender, age, and ethnicity.

#### 2.2.2. Stigma

Marlow and Wardle's [15] CSS is a 25-item scale, with items that tap awkwardness (e. g., “I would feel embarrassed discussing (type) cancer with someone who had it”), avoidance (e. g., “I would try to avoid a person with (type) cancer), severity (e. g., “(Type) cancer devastates the lives of those it touches), policy opposition (e. g., “More government funding should be spent on the care and treatment of those with (type) cancer,” reverse scored), personal responsibility (e. g., “If a person has (type) cancer it is probably their fault”), and financial discrimination (e. g., “It is acceptable for banks to refuse to make loans to people with (type) cancer”). The overall scale displayed good reliability (Cronbach's *α* = .837).

#### 2.2.3. Contact

For the cancer type participants were assigned to, participants were asked whether they have ever (1) lived with someone with (type) cancer, (2) had a family member with (type) cancer, (3) had a neighbour with (type) cancer, and (4) had a close friend with (type) cancer. Responses to these questions were summed and entered as covariates in the analysis.

#### 2.2.4. Empathy

Empathy was assessed using the empathic concern subscale of the Interpersonal Reactivity Index [16]. The subscale consists of 7 items (e. g., “When I see someone being treated unfairly, I sometimes do not feel very much pity for”, reverse scored) and displayed good reliability (Cronbach's *α* = .735). Responses to the empathic concern scale were averaged and entered as covariates in the analysis.

### 2.3. Data Analysis

Data were analyzed using jamovi, a free and open statistical platform. Means were calculated for each CSS subscale and submitted to an analysis of covariance (ANCOVA) with cancer type as a between-participant factor (5: lung, cervical, breast, skin, or bowel) and stigma as the dependent variable. Both the contact and empathy scores were entered as covariates. We used *p* < 0.05 as our level of significance.

## 3. Results

**Figure 1:**
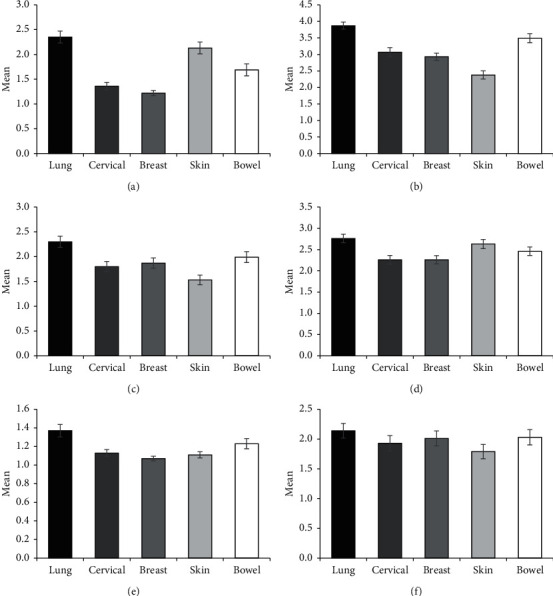
Mean scores on personal responsibility (a), severity (b), awkwardness (c), policy opposition (d), avoidance (e), and financial discrimination (f). Error bars represented ±1 standard error of the mean.

There was a main effect of cancer type for personal responsibility (F(4, 342) = 22.31, *p* < 0.001, Partial *η*^*2*^ = 0.207), severity (F(4, 342) = 19.79, *p* < 0.001, Partial *η*^*2*^ = 0.187), awkwardness (F(4, 342) = 5.82, *p* < 0.001, partial *η*^*2*^ = 0.064), policy opposition (F(4, 342) = 4.36, *p* < 0.002, partial *η*^*2*^ = 0.049), and avoidance (F(4, 342) = 5.34, *p* < 0.001, partial *η*^*2*^ = 0.059), but not financial discrimination (F(4, 342) = 0.96, *p* < 0.428, partial *η*^*2*^ = .011) (Figure 1).

For policy opposition, lung cancer attracted higher ratings than cervical, *t*(344) = 3.44, *p*=0.006 (Mean Difference: 0.501) and breast, *t*(344) = 3.493, *p* < 0.005 (Mean Difference: 0.502), but not skin, *t*(344) = 0.942, *p* < 0.880 (Mean Difference: 0.135), or bowel cancer, *t*(344) = 2.15, *p* < 0.200 (Mean Difference: 0.308). With respect to severity, lung cancer attracted higher ratings than cervical, *t*(344) = 4.61, *p* < 0.001 (Mean Difference: 0.797), breast, *t*(344) = 5.513, *p* < 0.001 (Mean Difference: 0.938), and skin, *t*(344) = 8.74, *p* < 0.001 (Mean Difference: 1.487), but not bowel cancer, *t*(344) = 2.20, *p* < 0.181 (Mean Difference: 0.374). For personal responsibility, lung cancer attracted higher ratings than cervical, *t*(344) = 6.82, *p* < 0.001 (Mean Difference: 0.995), breast, *t*(344) = 7.891, *p* < 0.001 (Mean Difference: 1.133), and bowel, *t*(344) = 4.64, *p* < 0.001 (Mean Difference: 0.664), but not skin cancer, *t*(344) = 1.55, *p* < 0.532 (Mean Difference: 0.222).

The results for avoidance and awkwardness were identical, with lung cancer attracting higher ratings than cervical (Avoidance: *t*(344) = 3.65, *p* < 0.003, Mean Difference: 0.242; Awkwardness: *t*(344) = 3.30, *p* < 0.009, Mean Difference: 0.497), breast (Avoidance: *t*(344) = 4.61, *p* < 0.001, Mean Difference: 0.301; Awkwardness: *t*(344) = 2.93, *p* < 0.029, Mean Difference: 0.774), and skin (Avoidance: *t*(344) = 4.04, *p* < 0.001, Mean Difference: 0.264; Awkwardness: *t*(344) = 5.23, *p* < 0.001, Mean Difference: 0.774), but not bowel cancer, (Avoidance: *t*(344) = 2.08, *p* < 0.229, Mean Difference: 0.136; Awkwardness: *t*(344) = 2.19, *p* < 0.211, Mean Difference: 0.314).

## 4. Discussion

The current study provides the first direct evidence that lung cancer attracts higher levels of stigma than other cancer types in Aotearoa New Zealand. Specifically, relative to most other cancer types, people rated lung cancer as more severe (e. g., ‘Once you've had lung cancer you are never ‘normal' again'), and were more likely to avoid someone with lung cancer, view interacting with them as more awkward, and tended to view people with lung cancer as being responsible for their condition (e. g., ‘A person with lung cancer is to blame for their condition'). Moreover, there was evidence of resistance to policies that would increase spending on lung cancer (e. g., ‘We have a responsibility to provide the best possible care for people with lung cancer, reverse scored). Given this latter finding, it is not surprising that Pharmac's decision to backtrack on funding Keytruda was met with relatively little outrage [10].

The degree of stigma observed in the current study is likely, in part, due to the strong link between lung cancer and smoking. Indeed, current or former smokers account for approximately 80% of lung cancer patients, leading people to view lung cancer as self-inflicted [17, 18]. Consistent with this view, participants in the current study rated people with lung cancer as being more responsible for their condition, compared to breast, cervical, and bowel cancer. Although skin cancer attracted comparable ratings for personal responsibility, lung cancer was still associated with higher levels of awkwardness and avoidance than skin cancer. One potential explanation of these latter findings is the impact of media campaigns designed to reduce smoking in Aotearoa New Zealand [19]. For example, the Smoking–Not Our Future campaign paired well-known people (e. g., All Blacks) with brief quotes/messages that aimed to make smokers less attractive, including “HELL NO! I WILL NEVER GO OUT WITH A SMOKER” and “I CAN'T STAND IT IF PEOPLE SMOKE AROUND ME” [20]. Although, as part of a much larger smoke-free campaign in Aotearoa New Zealand [21], these approaches appear successful, it is important to not neglect the fact that these campaigns may have negative impacts on how the public treats people with lung cancer [19].

The higher level of stigma lung cancer attracts may have especially important implications for Māori. It is well known that racism contributes to the health inequities experienced by Māori in Aotearoa New Zealand [22–24]. Data on cancer deaths also makes clear that lung cancer is somewhat of an outlier, with the disparity between Maori and non-Maori orders of magnitude larger than that for other cancer types [2, 4, 21]. One explanation for this collection of findings is intersectional stigma [25]. Intersectional stigma holds that occupying multiple stigmatised identities (i. e., being both Māori and having lung cancer) may not only have additive but potentially multiplicative effects on health [25]. Moreover, in keeping with our findings, identities that (1) deviate from social norms and (2) are associated with personal responsibility/victim blaming may have especially powerful effects, making lung cancer a prime target [25].

The current study is not without limitations. First, the majority of participants were university students (78%). Although this limits the generalisability of our findings, university students form a large proportion of the health workforce and many will likely occupy policy positions, making them an important population to assess. Second, we focused exclusively on individual-level stigma [26]. Stigma can also occur at a structural level and in the absence of individual-level discrimination [26]. For example, funding that prioritises research on other cancer types may make research on lung cancer less appealing to emerging scientists. Finally, as noted above, our study focused on a single stigmatised identity, leaving open questions about whether being both Māori and having lung cancer attracts higher levels of stigma. Future studies could utilise vignettes in which both ethnicity and cancer type can be manipulated to address the influence of intersectional stigma.

## 5. Conclusion

Lung cancer is the leading cause of cancer death in Aotearoa New Zealand but receives relatively little research funding and few donations [1]. Unfortunately, at least with respect to the cancer types included in the current study, lung cancer leads the way with respect to stigma, with patients not only attracting higher levels of blame but also higher levels of avoidance. Recently, in a promising step, following a cost-benefit analysis [27], the Health Research Council of New Zealand funded the very first trial of lung cancer screening in Aotearoa New Zealand [28]. Screening programmes, however, are not immune to stigma. Indeed, given that lung cancer patients are likely well aware of the stigma that exists [29], addressing stigma will be an important issue to address in screening programmes as it may influence those who choose to engage in such trials.

## Figures and Tables

**Table 1:** Demographic information for each condition/cancer type.

	Category	Lung	Cervical	Breast	Skin	Cervical
	*n*	72	66	70	70	71
	Mean age (SD)	22.9 (8.81)	26.0 (11.4)	25.1 (12.4)	23.8 (9.33)	23.7 (9.68)
	Student	59	46	55	55	58
	Nonstudent	13	20	15	15	15
Gender	Female	59	48	56	56	56
	Male	13	17	14	11	14
	Other	0	1	0	3	1
Ethnicity	NZ European	45	52	52	52	54
	Māori	7	6	8	5	6
	Asian	4	3	2	6	7
	Pacific	3	0	1	0	0
	Other	13	5	7	7	4

## Data Availability

The data used to support the findings of this study are available from the corresponding author upon request.
